# Activity of glucose oxidase functionalized onto magnetic nanoparticles

**DOI:** 10.1186/1477-044X-3-1

**Published:** 2005-03-11

**Authors:** Gilles K Kouassi, Joseph Irudayaraj, Gregory McCarty

**Affiliations:** 1 Department of Agricultural and Biological Engineering, The Pennsylvania State University, University Park, PA 16802, USA; 2 Department of Engineering Sciences and Mechanics, The Pennsylvania State University, University Park, PA 16802, USA

## Abstract

**Background:**

Magnetic nanoparticles have been significantly used for coupling with biomolecules, due to their unique properties.

**Methods:**

Magnetic nanoparticles were synthesized by thermal co-precipitation of ferric and ferrous chloride using two different base solutions. Glucose oxidase was bound to the particles by direct attachment via carbodiimide activation or by thiophene acetylation of magnetic nanoparticles. Transmission electron microscopy was used to characterize the size and structure of the particles while the binding of glucose oxidase to the particles was confirmed using Fourier transform infrared spectroscopy.

**Results:**

The direct binding of glucose oxidase via carbodiimide activity was found to be more effective, resulting in bound enzyme efficiencies between 94–100% while thiophene acetylation was 66–72% efficient. Kinetic and stability studies showed that the enzyme activity was more preserved upon binding onto the nanoparticles when subjected to thermal and various pH conditions. The overall activity of glucose oxidase was improved when bound to magnetic nanoparticles

**Conclusion:**

Binding of enzyme onto magnetic nanoparticles via carbodiimide activation is a very efficient method for developing bioconjugates for biological applications

## Background

The immobilization of biomolecules onto insoluble supports is an important tool for the fabrication of a diverse range of functional materials or devices [1]. Enzyme immobilization for example, is a desired biological procedure because of the possible application of immobilized enzymes in continuous operations, product purification, and catalyst recycling [2]. Furthermore, immobilization provides many advantages such as enhanced stability, easy separation from reaction mixture, possible modulation of the catalytic properties, and easier prevention of microbial growth [3].

In the last decade, nanosize materials have been widely used as support for this purpose. Among these materials, magnetic nanoparticles are very popular when used in conjunction with biological materials including proteins, peptides, enzymes [4-9] antibodies and nucleic acids [8], because of their unique properties [4-9]. The ability to tract magnetically labeled entities or target organelles using magnetic force offer the opportunity to conduct biological operations with increased specificity.

Magnetite (Fe_3_O_4_) are biocompatible superparamagnetic materials that have low toxicity and strong magnetic properties [5]. They have been widely used for *in *vivo examination including magnetic resonance imaging, contrast enhancement, tissue specific release of therapeutic agents, hyperthermia [10,11], magnetic field assisted radionucleide therapy [11], as well as *in *vitro binding of proteins and enzymes [4-8].

Magnetite nanoparticles have been used as support material for binding of enzymes including yeast alcohol dehydrogenase [4] and lipase [5] directly via carbodiimide activation. This method brought about considerable promise because of its simplicity and efficiency. Recently, γ-Fe_2_O_3_magnetic nanoparticles were used for binding *Candida rugosa *lipase after acetylation of thiophene functionalized nanoparticles, or through nitroso-derivative formed on the surface of the particles by reacting nitroso tetrafluoroborate in methylene chloride. Both methods and more effectively lipase immobilized on acetylated nanoparticles exhibited long term stability. Glucose oxidase (GOX, β-D-glucose oxygen 1-oxidoreductase, EC 1.1.3.4) is a homodimer flavoprotein containing two active sites per molecule [12,13]. It catalyses the oxidation of β-D-glucose to gluconic acid, concomitant with the reduction of oxygen to hydrogen peroxide. Glucose oxidase has been used to test various types of enzyme immobilization, and is the most commonly studied in the construction of biosensors for glucose assay development [12,14,15]. A more recent study [16] examined the activity of cholesterol oxidase activity using carbodiimide activation.

Here, we report the stability and enzymatic activity of glucose oxidase immobilized onto Fe_3_O_4 _magnetic nanoparticles using two binding methods, the direct binding via carbodiimide activation of amino functionalized particles and binding to thiophene-functionalized acetylated nanoparticles. A comparison of the stability and activity of glucose oxidase immobilized to magnetite using different protocols will lay the foundation for magnetic immunoassays. The size and structure of the nanoparticles were characterized using Transmission electron microscopy (TEM) and Fourier Transform Infrared (FTIR) spectroscopy, respectively. The stability, activity, and kinetic behavior of bound glucose oxidase were also examined.

## Materials and methods

Glucose oxidase (specific activity 200 units/mg protein) from *Aspergillus *niger was purchased from VWR international (Pittsburgh, USA). Carbodiimide-HCl (1-ethyl-3-(3-dimethyl-aminopropyl), ammonium hydroxide, sodium hydroxide, acetic anhydride, glucose, bovine serum albumin (BSA), iron (II) chloride tetrahydrate 97 % and iron (III) chloride hexahydrate 99%, were obtained from Sigma-Aldrich St Louis (USA). 11-bromoundecanoic acid was obtained from TCI America Portland, USA. The Biorad Protein Assay Reagent Concentrate was purchased from Biorad Laboratories (Hercules, CA). Thiophene-2-thiolate was obtained from Alfa Aesar MA, USA. Iodine was obtained from Mallinckrodt Kentucky, USA, and acetonitrile was obtained from EMD Chemicals (New Jersey, USA). Sodium phosphate monohydrate and potassium phosphate dihydrate were acquired from EM Science (New Jersey, USA). Sodium carbonate was obtained from Orion Research Inc. (Beverly, USA). Magnetic nanoparticles (Fe_3_O_4_) were prepared by chemical co-precipitation of Fe^2+ ^and Fe^3+ ^ions in a solution of ammonium hydroxide (magnetic nanoparticles I or Fe_3_O_4 _I), or sodium hydroxide (magnetic nanoparticles II or Fe_3_O_4 _II) followed by a treatment under hydrothermal conditions [4,5]. Iron (II) chloride and iron (III) chloride (1:2) were dissolved in nanopure water at the concentration of 0.25 M iron ions and chemically precipitated at room temperature (25°C) by adding NH_4_OH solution (30%) or NaOH 3 M at a pH 10. The precipitates were heated at 80°C for 35 min under continuous mixing and washed 4 times in water and several times in ethanol. During washing, the magnetic nanoparticles were separated from the supernatant using a magnetic separator of strength greater than 20 megaoersted (MOe). The particles were finally dried in a vacuum oven at 70°C. The dried particles exhibited a strong magnetic attraction.

Magnetic nanoparticles I (50 mg) produced, using a solution of ammonium hydroxide were added to 1 mL of phosphate buffer (0.05 M. pH 7.4). After adding 1 mL of carbodiimide solution (0.02 g/mL) in phosphate buffer (0.05 M. pH 7.4), the mixture was sonicated for 15 min. Following the carbodiimide activation, 2 mL of glucose oxidase (1000 units /mL) was added and the reaction mixture was sonicated for 30 min at 4°C in a sonication bath. The magnetic nanoparticles were separated from the mixture using a magnetic separator. The precipitates containing Fe_3_O_4 _nanoparticles I and Fe_3_O_4 _bound glucose oxidase (GOX-Fe_3_O_4 _I) were washed with phosphate buffer pH 7.4 and 0.1 M Tris, pH 8.0, and then used for activity and stability measurements. NaCl was added to enhance the separation of the magnetic nanoparticles [4].

A second functionalization protocol using a modification of the strategy adopted to immobilize *Candida rugosa *lipase on the γ-Fe_2_O_3_[12] was implemented (Magnetic nanoparticles II). Briefly, 1.5 g Fe_3_O_4 _was added to 5 g of 11-bromoundecanoic acid dissolved in 15 mL of ethanol. 11-bromoundecanoic acid was covalently linked to the nanoparticles surfaces by heating the mixture with microwave irradiation for 10 min. Functionalization of the particle was achieved through nucleophilic substitution with the 2-thiophene thiolate. In practice, 2-thiophene thiolate (7 mL) was added to the mixture containing the particles and heated in microwave for 5 minutes. The mixture was washed with ethanol and transferred in a round bottom flask. Acetic anhydride (4 mL) and 34.6 mL of iodine (0.01N) were successively added to the particles and agitated. The mixture was heated for 1 h under reflux condition [17]. The particles were washed several times with water, once with 10% sodium carbonate solution and finally with ethanol. The acetylated particles were reacted directly with the enzyme covalently linked to the particles via C = N bond [11].

**Figure 1:**
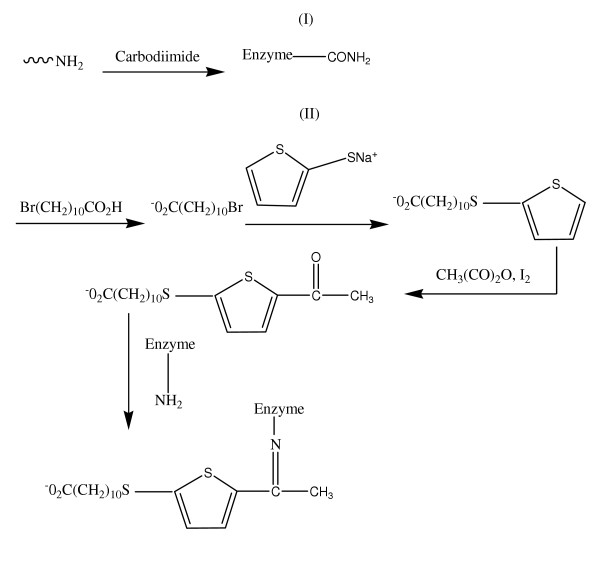
Description of GOX attachment procedures. (I) procedure employed for GOX-Fe_3_O_4 _I attachment, (II) thiophene functionalization and the acetylation of particles for GOX-Fe_3_O_4 _II attachment.

For the attachment of glucose to nanoparticles, 2 mL of the GOX solution (1000 units/mL) was added to 50 mg of functionalized magnetic nanoparticles in a test tube and sonicated at 15°C for 3 h. The supernatant containing unbound enzymes was separated from the magnetic nanoparticles using the magnetic separator, and the enzymes bound to magnetic nanoparticles were then used for activity determination. A schematic of the procedures used for both attachments are presented in Figure 1. The amount of protein in the supernatant was determined by a colorimetric method at 595 nm with the Biorad Protein Assay Reagent Concentrate using bovine serum albumin (BSA) as the protein standard. The amount of bound enzymes was calculated from:

*A *= (*C*_*i *_- *C*_*s*_)* *V *    (1)

Where *A *is the amount of bound enzyme, *C*_*i *_and *C*_*s *_are the concentration of the enzyme initially added for attachment and in the supernatant, respectively (mg/mL) and *V *is the volume of the reaction medium (mL). The size of Fe_3_O_4 _magnetic nanoparticles, GOX-Fe_3_O_4 _I and GOX-Fe_3_O_4 _II were characterized by transmission electron microscopy (TEM, JEM 1200 EXII, JEOL) and structure by FTIR spectroscopy (Biorad FTS 6000, Cambridge, MA). The samples for TEM analysis were prepared as follows: a drop of magnetic nanoparticles was dispersed in nanopure water. The resulting solution was sonicated for 4 min to obtain better particle dispersion. A drop of the dispersed solution was then deposited onto a copper grid and dried overnight at room temperature. The binding of GOX onto the magnetic nanoparticles was investigated using FTIR spectroscopy. Samples for FTIR analysis were prepared in phosphate buffer pH 7.4. The activity of bound GOX was determined by measuring the initial rate of formation of hydrogen peroxide at a given temperature following the formation of a red quinoneimine dye. The principle of enzymatic determination of the activity of glucose oxidase is described as follows: Glucose is oxidized by glucose oxidase to gluconate and hydrogen peroxide. Phenol + 4-AAP, in the presence of peroxidase (POX), produces a quinoneimine dye that is measured at 500 nm using a Beckman Du Spectrometer to provide an absorbance that is proportional to the concentration of glucose in the sample. The reaction is described as follows:





The activity of glucose oxidase was measured as follows. An assay mixture was prepared by mixing 500 U of horseradish peroxidase, 0.015 mmol of 4-aminoantipyrine (4-AAP), 0.025 mmol of phenol and 5 mmol of glucose in 50 mL of phosphate buffer solution (0.05 M. pH 7.4) to result in a glucose concentration of 0.1 M. To start the enzymatic reaction, 2 mL of the assay solution was added to 15 mL centrifuge test tubes containing GOX-Fe_3_O_4 _and mixed by vortex. A solution of free GOX of the same molar concentration was used to evaluate the activity of the free enzyme for comparison. The solution was incubated at various temperatures (37–80°C) at specific intervals of time (30 min) and the supernatant was separated from GOX-Fe_3_O_4 _using a magnetic separator. 10 μL aliquots of the supernatant were then taken for determining the concentration of hydrogen peroxide following the procedure by Trinder [18]. The activity of the enzyme can be calculated using the following equation:



Where *ABS *sample denotes the absorbance of the sample, *ABS Std *is the absorbance of the standard solution, and C the concentration of glucose in the sample.

The effect of temperature on the free GOX, GOX-Fe_3_O_4 _I and GOX-Fe_3_O_4 _II was estimated by determining the concentration of glucose in the sample at various temperatures. A solution of the assay mixture was added to the various centrifuge test tubes containing bound or free enzymes. The samples were stored in a water bath at specific temperatures (37, 50, 60, 70 and 80°C) and the absorbance was monitored at fixed time intervals to determine the glucose content. The effect of pH on GOX was monitored by measuring the initial rate of glucose oxidation by glucose oxidase in different phosphate and carbonate buffer solutions of pH (5–10) at 25°C.

The thermal stability of free GOX, GOX-Fe_3_O_4 _I, and GOX-Fe_3_O_4 _II were determined by measuring the residual activity of the enzyme at 25°C, after being exposed to different temperatures (37–80°C) in phosphate buffer (0.05 M, pH 7.4) for 30 min. Aliquots of the reacting solutions were taken at periodic intervals (every 30 min for 6 h) and assayed for enzymatic activity as described above. The first-order inactivation rate constant, *k *was calculated from the equation:

ln *A *= ln *A*_0 _- *kt *    (5)

where *A*_0 _is the initial activity, *A *is the activity after time t (min) and *k *is the reaction constant.

The storage stability was examined by measuring the change in the concentration of glucose at room temperature at different time intervals (4 days). Test tubes with samples of GOX-Fe_3_O_4 _I, GOX-Fe_3_O_4 _II, or free GOX solutions were stored at 25°C in phosphate buffer (0.05 M. pH 7.4) for 33 days. Thereafter, 3 mL of the assay solution was added, and the residual activity of GOX was assayed.

The kinetic parameters of free GOX, GOX-Fe_3_O_4 _I and GOX-Fe_3_O_4 _II, *K*_m _and *V*_max _were determined by measuring the initial rates of glucose oxidation (0.2–1 mM) by glucose oxidase (0.25 mg/mL) in phosphate buffer (pH 7.4) at 25°C.

## Results and discussion

**Figure 2:**
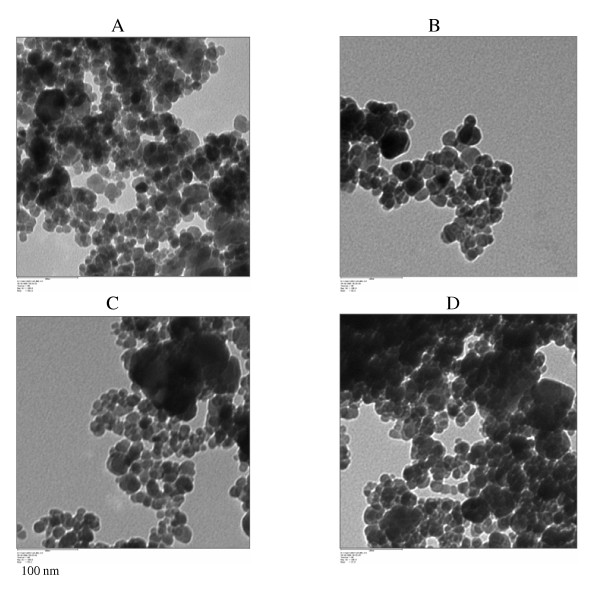
Transmission electron micrographs of Fe_3_O_4 _magnetic nanoparticles I (A), Fe_3_O_4 _magnetic nanoparticles II (B), GOX-Fe_3_O_4 _I (C), and GOX-Fe_3_O_4 _II (D).

**Figure 3:**
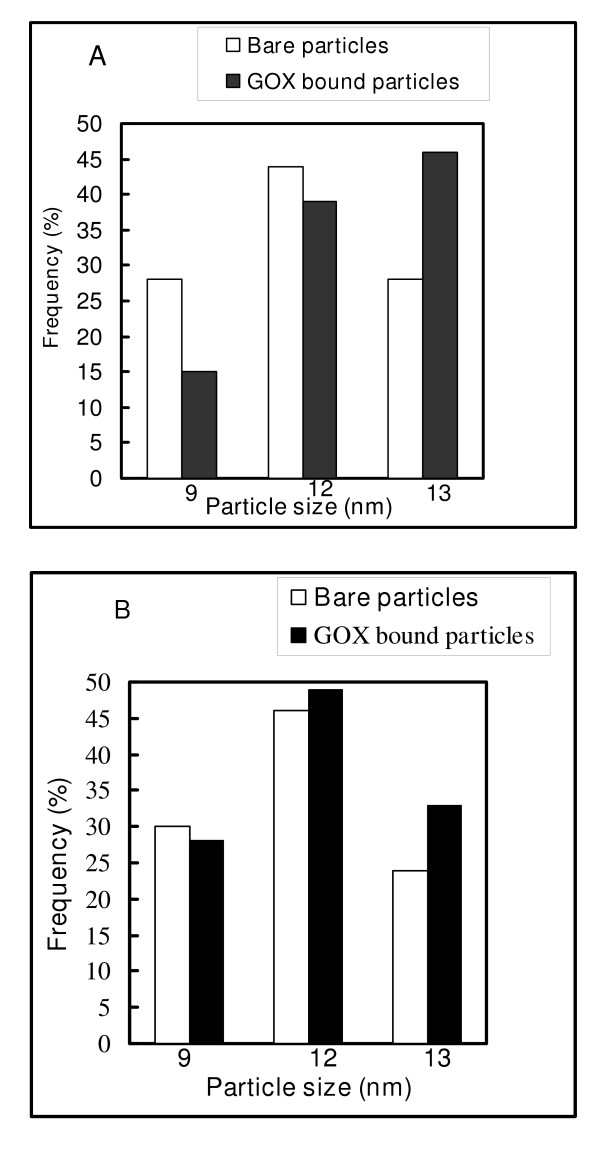
Distribution of particles on the electron micrographs of Fe_3_O_4 _magnetic nanoparticles I and GOX-Fe_3_O_4 _I (A), and Fe_3_O_4 _magnetic nanoparticles II and GOX-Fe_3_O_4 _II (B).

Synthesis of magnetic nanoparticles with equivalent amounts of ferric and ferrous chlorides resulted in 66% of magnetic nanoparticles I and 73% of magnetic nanoparticles II. These yields suggested that the synthesis using NaOH is more advantageous for large scale production of magnetic nanoparticles. "Bare" Fe_3_O_4 _I and Fe_3_O_4 _II, and their GOX bound counterparts shown in the TEM micrographs in Figures (2A, B, C and 2D), respectively reveal that the particles are fine and spherical. The sizes of the particles of each sample were evaluated from 2 different TEM images. The diameter was in the range between 9 and 13 nm. The coefficient of variation between different measurements was less than 7%. There was no significant change in the size of the 'bare" particles and GOX bound particles. However, signs of agglomeration of the particles were visible in the samples. The agglomerates were not considered in the examination of the size distribution of the magnetic particles because of the assumption that agglomerated particles do not describe the original size of the particles. Figure 3 shows the distribution of the particles sizes. Fe_3_O_4_nanoparticles 14.5 mg/mL corresponding to GOX/Fe_3_O_4 _weight ratios of 0.2 was used for the binding process. The amount of unbound enzyme was determined by assaying the protein content in the supernatant. The amount of enzymes bound to the magnetic nanoparticles in each binding procedure is given in table 1. Average binding efficiencies of 38.4 units/mg particles for GOX-Fe_3_O_4 _I and 27.6 units/mg particles for GOX-Fe_3_O_4 _II were noted. The overall percentage of binding efficiency was between 94 and 100% for GOX-Fe_3_O_4 _I and 66 to 72% for GOX-Fe_3_O_4 _II. These results show that the binding was successful in both cases, particularly with Fe_3_O_4 _I which uses the carbodiimide activation. The percentage of binding of GOX to Fe_3_O_4_II was significant but still below the level obtained through carbodiimide activation with the Fe_3_O_4 _I. However, despite the difference in the level of GOX bound, the two procedures adopted were successful.

**Table 1:** Summary of binding efficiencies of enzyme-functionalized systems (n = 9).

	Bound enzymes (units/mg nanoparticles)
GOX-Fe_3_O_4 _I	38.4 ± 0.8
GOX-Fe_3_O_4 _II	27.6 ± 0.6

**Figure 4:**
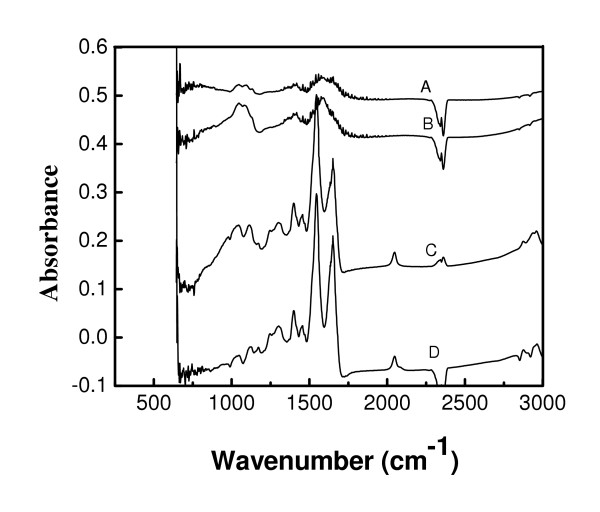
FTIR spectra of Fe_3_O_4 _magnetic nanoparticles I (A) and Fe_3_O_4 _magnetic nanoparticles II (B), GOX-Fe_3_O_4 _I (C), and GOX-Fe_3_O_4 _II (D).

The FTIR spectra of magnetic Fe_3_O_4 _I, Fe_3_O_4 _II, GOX-Fe_3_O_4 _I and GOX-Fe_3_O_4 _II are shown in Figure 4(A, B, C, D), respectively. The characteristic bands of proteins at 1541 and 1645 cm^-1 ^assigned to amide I and amide II, respectively were visible in the spectra of GOX-Fe_3_O_4 _I and GOX-Fe_3_O_4 _II. These peaks are associated with two sharp peaks in the region 1420-1300 cm^-1 ^typical of carboxylate groups, from the enzyme. A weak peak at 1900 cm^-1 ^appeared in the spectra of GOX-Fe_3_O_4 _I and GOX-Fe_3_O_4 _II and could be assigned to the C-O bonds in the enzyme molecule. These peaks were absent in the spectra of Fe_3_O_4 _I and Fe_3_O_4 _II. In the 1100-1000 cm^-1 ^region in all spectra appeared a characteristic adsorption spectra typical to phosphate ion that can be assigned to the phosphate buffer used in the samples preparation. The occurrence of negative peaks in the spectra of Fe_3_O_4 _I, and GOX-Fe_3_O_4 _II is possibly due to the reduced amount of phosphate in comparison to the amount of phosphate used to background subtraction. The characteristic peaks of proteins found in the spectra of GOX-Fe_3_O_4 _I and GOX-Fe_3_O_4 _II were not visible in the spectra of Fe_3_O_4 _I and Fe_3_O_4 _II, indicating enzyme attachment onto the particles. The amino groups on the surface of the particles resulted from the use of concentrated ammonia solution during the co-precipitation of Fe^2+ ^and Fe^3+ ^as demonstrated by [5] and [19].

**Figure 5:**
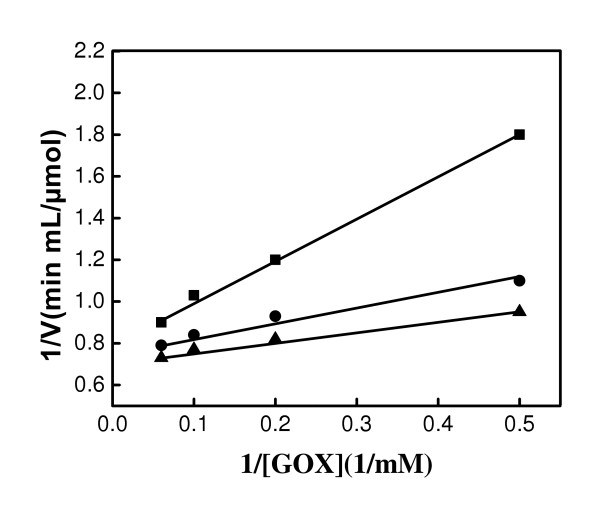
Double reciprocal plots of the initial rates of free GOX (■), GOX-Fe_3_O_4 _I (▲) and GOX-Fe_3_O_4 _II (●) at pH 7.4, from experimental data.

The most important aspect of this study is related to the retention of biocatalytical activity of GOX after binding to magnetic nanoparticles. The amount of bound GOX was estimated using the UV-vis spectrophotometer and the catalytic activity of free and bound GOX was compared. Kinetic parameters (*V*_*m *_and *K*_*m*_) were estimated from the double reciprocal plots of the initial rates of glucose oxidation by GOX. The double reciprocal plots are presented in Figure 5. The Michaelis-Menten constants *V*_max _and *K*_m _for GOX are shown in Table 2. *V*_max _of free GOX, GOX- Fe_3_O_4 _I and GOX-Fe_3_O_4 _II were 0.731, 0.803, and 0766 μmol/min mL and, the corresponding *K*_m _values were, 0.383, 0.208, and 0.237 mM, respectively. The *V*_max _value of GOX immobilized on magnetic nanoparticles (I and II) was higher than that of the free enzyme. The highest *V*_max _was obtained with GOX-nanoparticles I. Since a low *K*_m _indicates a high degree of affinity of the enzyme to substrate [5], calculations showed that the affinity of the enzyme to the substrate increased in the order of free GOX, GOX-Fe_3_O_4 _II and GOD-Fe_3_O_4 _I, respectively. The high affinity of the enzyme to the substrate may be explained by a favorable change in the structural organization of the enzyme due to the immobilization procedure [20]. Consequently, the active sites of the enzymes could be more readily available for enzymatic interactions.

**Table 2:** Kinetic parameters of free GOX, GOX-Fe_3_O_4 _I, and GOX-Fe_3_O_4 _II determined from the double reciprocal plots.

	V_max _(μmol/min mL)	*K*_m _(mM)
	
Free GOD	0.731	0.383
GOD-Fe_3_O_4 _I	0.803	0.208
GOD-Fe_3_O_4 _II	0.766	0.237

**Figure 6:**
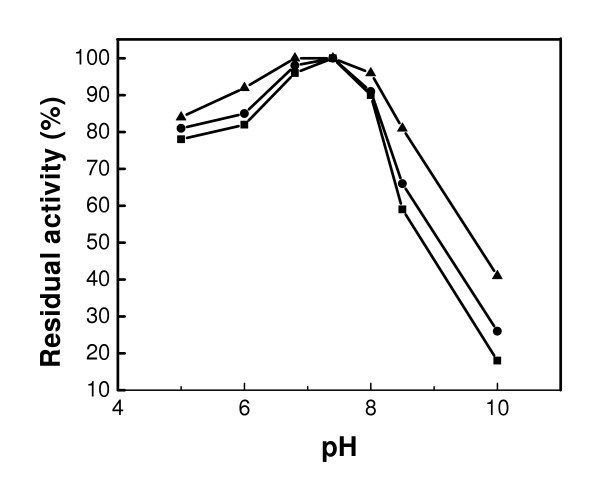
Effect of pH on the activities of free GOX (■), GOX-Fe_3_O_4 _I (▲) and GOX-Fe_3_O_4 _II (●).

The effect of pH on the activities of the free and bound GOX was investigated in the pH range of 5–10 at 25°C (Figure 6). Each data point was the average of two measurements. The coefficient of variation between measurements was between 2 and 5%. In the pH range between 6 and 7.4, the enzyme activity increased in all the systems. However, the enzyme activity was higher in GOX-Fe_3_O_4 _I than in GOX-Fe_3_O_4 _II and free GOX. The activity reached 100% at pH 7.4, and decreased above this pH value to 43% for GOX-Fe_3_O_4 _I, and to 26% for GOX-Fe_3_O_4 _II and 9% for the free GOX at pH 10. The free GOX experienced a more severe loss in activity, while GOX-Fe_3_O_4 _I retained greater activity as the pH increased. In this system, the binding process occurred directly upon activation of the particle surface using carbodiimide, while the binding of GOX-Fe_3_O_4 _II involved a C = N bond formed on the acetylated thiophene. It can be argued that the direct binding between the protein and the amino bond in the former case exhibited a greater resistance to a medium with higher alkalinity. This medium appeared even more constraining to the free enzyme and placed the enzyme in an electrostatic state that might affect the activity.

**Figure 7:**
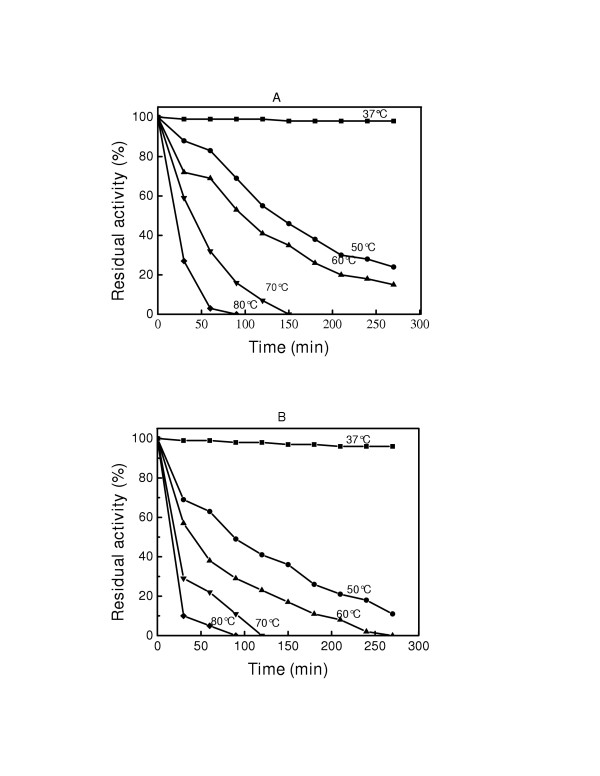
Effect of temperature on the activity of GOX-Fe_3_O_4 _I (A) and GOX-Fe_3_O_4 _II (B) at pH 7.4. The samples were stored at 37, 50, 60, 70 and 80°C for 30 min and the activities were measured at 25°C.

**Figure 8:**
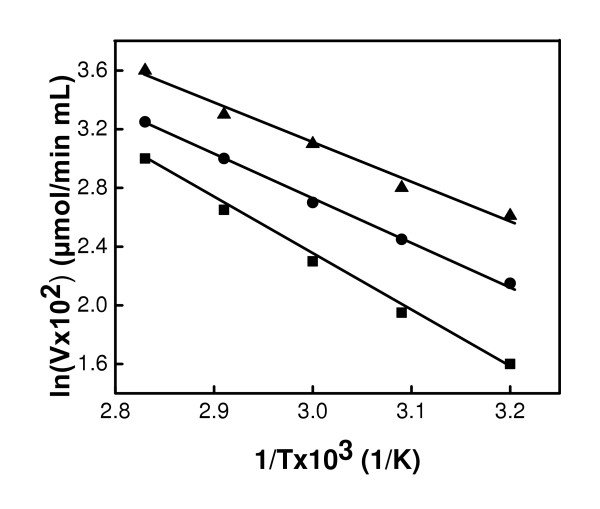
Arrhenius plots of the initial oxidation rates of glucose by free GOX (■), GOX-Fe_3_O_4 _I (▲) and GOX-Fe_3_O_4 _II (●) for samples at 37, 50, 60, 70, and 80°C.

The effect of temperature on the activity of free GOX was examined by measuring its relative activity when stored at various temperatures. Figure 7a and 7b shows the effect of temperature on GOX-Fe_3_O_4 _I and GOX-Fe_3_O_4 _II at various temperatures. Each data point represents the average of duplicate measurements (coefficient of variation was less than 6 %). It can be observed that at 37°C, the enzyme retained its activity for about 80 minutes before showing a slight decrease. At 50, 60, 70 and 80°C the activity decreased as the temperature increased in both systems. In GOX- Fe_3_O_4_, the remaining activity was 23% at 50°C and 15% at 60°C after 270 min. For this time period and duration, the remaining activities were 9% and 0%, respectively for GOX-Fe_3_O_4 _II. A similar trend was observed at 70 and 80°C with a more drastic loss of activity from the GOX-Fe_3_O_4 _II protocol. Loss of activity occurred more rapidly in GOX-Fe_3_O_4 _II indicating that direct binding through carbodiimide activation provides a greater thermal stability to GOX. The effect of temperature on the activities of free, GOX-Fe_3_O_4 _I, and GOX-Fe_3_O_4 _II at pH 7.4 are presented in the Arrhenius plots (Figure 8). The activation energies were calculated as 1.4, 0.9, and 1.1 kJ/mol for free-GOX, GOX-Fe_3_O_4 _I, and GOX-Fe_3_O_4 _II, respectively. These results show that the unbound enzyme has the highest activation energy, while GOX-Fe_3_O_4 _I had the lowest. The low activation energy of GOX associated with binding to magnetic nanoparticles suggests that the energy requirement on the surfaces of the nanoparticles for enzymatic activity is reduced. Table 3 shows the inactivation rates constants (*k*) at 50, 60 70, and 80°C. The rate constants increased with increasing temperature in the order GOX-Fe_3_O_4 _I, GOX-Fe_3_O_4 _II and free GOX. Here again, binding GOX to magnetic nanoparticles minimized structural denaturation due to heat treatment. Covalent binding was expected to provide the enzyme with the protection against structural denaturation due to the unfavorable solvent-protein interactions, and thus result in activation effect [21], a possible reason for a better activity of the bound enzyme compared with the free enzyme after heat treatment. GOX-Fe_3_O_4 _II had a lower stability at higher temperatures compared to GOX-Fe_3_O_4 _I. The reason for this difference could reside in the stability of the binding, since the binding methods are so far the major difference between these systems. Indeed with GOX-Fe_3_O_4 _I, the binding of the enzyme occurred through the amino groups on the surfaces of the particles and the carboxylic groups of proteins in the enzymes [5] which is a natural way for protein binding, while in the case of GOX-Fe_3_O_4 _II the N atom to which the enzyme is attached shared a double bond with the carbon atom which is less stable than the amide bond.

**Table 3:** Inactivation rate constants (*k*) of the free-GOX, GOX-Fe_3_O_4 _I, and GOX-Fe_3_O_4_II at various temperatures.

Temperature (°C)	Free-GOX *k *(min^-1^)	GOX-Fe_3_O_4 _I *k *(min^-1^)	GOX-Fe_3_O_4 _II *k *(min^-1^)
50	3.42. 10^-2^	7.10.10^-4^	2.87.10^-3^
60	9.36.10^-2^	1.49.10^-3^	3.50.10^-2^
70	2.81.10^-1^	6.24.10^-2^	1.92.10^-2^
80	9.3210^-1^	4.52.10^-2^	5.46.10^-1^

**Figure 9:**
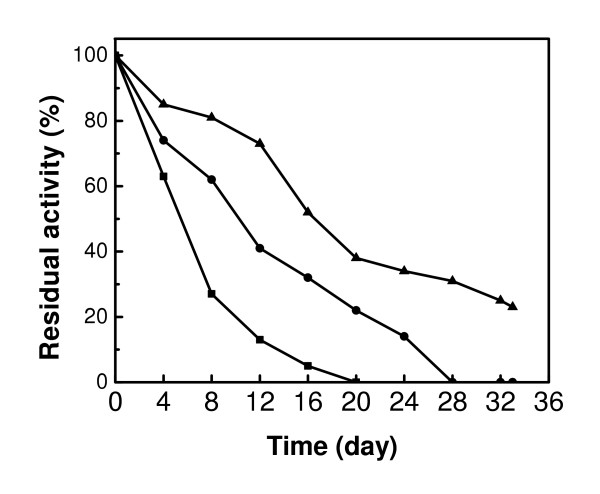
Storage stability of free GOX (■), GOX-Fe_3_O_4 _I (▲), and GOX-Fe_3_O_4 _II (●) measured at a pH of 7.4 at 25°C.

Loss of storage stability is a major concern in enzyme preservation. The storage stability of the enzyme was examined for 33 days. Figure 9 shows the storage stabilities of free GOX, GOX-Fe_3_O_4 _I, and GOX-Fe_3_O_4 _II at 25°C at pH 7.4. Each data point was the average of duplicate measurements (coefficient of variation of the measurements was between 1 and 5%). The activity decreased with time in all the systems. Total loss of activity was observed after 20 days for the free GOX and 28 days for GOX-Fe_3_O_4 _II while GOX-Fe_3_O_4 _I retained 26% activity after 33 days of storage under identical conditions. The stability of the enzyme was found to improve upon binding to the magnetic nanoparticles but the most significant improvement in stability was observed for the GOX-Fe_3_O_4 _I nanoparticle complex. The fixation on the surface of the magnetic nanoparticles has been a tangible argument supporting the prevention of auto-digestion of the enzyme and lysis, and the subsequent conservation of its activity [4]. This argument supports our results and justifies the long term stability of GOX-nanoparticles I and GOX-nanoparticles II over the free GOX. The efficiency of binding of GOX via carbodiimide activation over the binding by thiophene acetylation may be attributed to the potential of carbodiimide to activate the carboxylic acid side chains partially buried at the surface or in active sites of the enzyme, as well as the amino groups on the nanoparticles, favoring the formation and the stability of the amide bond [22]. This may explain why the amount of bound enzymes is higher in the binding via carbodiimide than with the thiophene acetylation. Furthermore, carbodiimide might cause cross-linking of the enzyme providing a better stability to its quaternary structure [23] and a subsequent improvement in stability.

## Conclusion

Magnetic nanoparticles were synthesized by thermal co-precipitation of ferric and ferrous chlorides using two different base solutions. GOX was bound to the particles by direct attachment via carbodiimide activation and chemically via covalent attachment onto thiophene acetylated magnetic nanoparticles. Confirmation of the binding was demonstrated by FTIR spectroscopy and the sizes of the particles were characterized by TEM. The direct binding of GOX via carbodiimide activation was more effective and resulted in binding efficiency in the range between 94–100% while the binding efficiency was only between 66–72% for the GOX-Fe_3_O_4 _II complex. Kinetic and stability studies showed that the enzyme activity was more preserved upon binding onto the nanoparticles when the complex was subjected to thermal and pH variations. This study shows that binding onto magnetic nanoparticles can allow the enzyme to acquire the conformational and structural arrangement for a better activity and stability, and suggests that binding of enzyme onto magnetic nanoparticles via carbodiimide activation was efficient for creating bioconjugates for a variety of applications in health and food safety.

## Authors' contributions

Drs. Gilles Kouassi and Joseph Irudayaraj were the primary authors. They were responsible for the concept and experimental plan of the article. Dr Gregory MacCarty was the secondary author and contributed to the overall effort.
